# Contribution of Crk Adaptor Proteins to Host Cell and Bacteria Interactions

**DOI:** 10.1155/2014/372901

**Published:** 2014-11-25

**Authors:** Narcisa Martinez-Quiles, Leigh Ann Feuerbacher, María Benito-León, Philip R. Hardwidge

**Affiliations:** ^1^Department of Microbiology, Medical School, Complutense University of Madrid, Avenida de la Complutense sn, 28040 Madrid, Spain; ^2^Kansas State University, 1600 Denison Avenue, Manhattan, KS 66506, USA

## Abstract

The Crk adaptor family of proteins comprises the alternatively spliced CrkI and CrkII isoforms, as well as the paralog Crk-like (CrkL) protein, which is encoded by a different gene. Initially thought to be involved in signaling during apoptosis and cell adhesion, this ubiquitously expressed family of proteins is now known to play essential roles in integrating signals from a wide range of stimuli. In this review, we describe the structure and function of the different Crk proteins. We then focus on the emerging roles of Crk adaptors during Enterobacteriaceae pathogenesis, with special emphasis on the important human pathogens *Salmonella*, *Shigella*, *Yersinia*, and enteropathogenic *Escherichia coli*. Throughout, we remark on opportunities for future research into this intriguing family of proteins.

## 1. Introduction

The product of the oncogene* v-Crk* was identified in 1988 due to its capacity to transform fibroblasts and to induce tumors in chickens and was named CT10 (chicken tumor virus number 10) regulator of kinase, Crk [1, 2]. The demonstration that the v-Crk protein was able to increase significantly the tyrosine-phosphorylation of proteins in cells without having any intrinsic kinase activity itself was a breakthrough that contributed to the description of the SH2-phosphotyrosine interaction module which functions in cell signaling. Today, the v-Crk cellular homologs (c-Crk) CrkI and CrkII, together with the paralog Crk-like (CrkL) protein, are categorized as adaptor proteins.* CrkL* was first cloned as a gene implicated in chronic myeloid leukemia (CML) [3]. Soon afterward, CrkL was found to be the major phosphoprotein detected in CML cells [4]. The discovery of Crk adaptor proteins represents a milestone in the signal transduction field [5, 6]. These proteins were first implicated in signaling during apoptosis [7] and cell adhesion and migration (reviewed in [8, 9]). Currently, this ubiquitously expressed family of proteins is known to play essential roles in integrating signals from a wide variety of cellular processes, including those more specific to the immune system [10]. In this review, we focus on the emerging roles of Crk adaptors during bacterial pathogenesis.

## 2. Knockout Mouse Models

CrkI and CrkII are alternatively spliced products from a gene locus located on human chromosome 17p13.3 [11, 12]. CrkL is a paralog encoded by a distinct gene located on human chromosome 22q11.21 [3]. CrkL shares high sequence homology with CrkII, bringing into question whether the two proteins have overlapping functions and the ability to compensate for one another. Unfortunately, the different Crk knockout (K.O.) mouse models have not provided a simple answer to this important question. The first Crk K.O. mouse model was generated by gene trap insertional mutagenesis, resulting in the elimination of CrkII but not of CrkI [13]. These animals developed normally, indicating that CrkI can compensate for the absence of CrkII. Much later, complete ablation of both CrkI and CrkII was achieved and it was observed that most animals died perinatally, mainly due to vascular, cardiac, and craniofacial development defects [14].

There are two different CrkL K.O. mouse models. One was used to develop a transgenic mouse bearing the* BCR-ABL* fusion gene [15]. Unexpectedly, the CrkL K.O., as well as the transgenic animals, developed leukemia and lymphoma. Furthermore, CrkII was found to be tyrosine phosphorylated and associated with the Bcr-Abl chimeric protein in these cells, suggesting that CrkII replaced CrkL tumorigenic functions. CrkL maps to the 22q11.2 chromosomal region frequently deleted in heterozygosis in humans afflicted with the DiGeorge/velocardiofacial syndrome (now referred to as 22p11DS, Orpha number ORPHA567). The second CrkL K.O. mouse model was developed by the research group of Dr. Akira Imamoto (University of Chicago) and had severe developmental alterations that recapitulated some of the defects typically found in DiGeorge syndrome, such as defects of the cranial and cardiac neural crest derivatives, including a thymus aplasia [16] that causes immunodeficiency [17]. Importantly, MEFs were produced [18] that, much later, allowed investigation into how CrkL contributes to pedestal formation by enteropathogenic* E. coli*.

## 3. Structure

**Figure 1:**
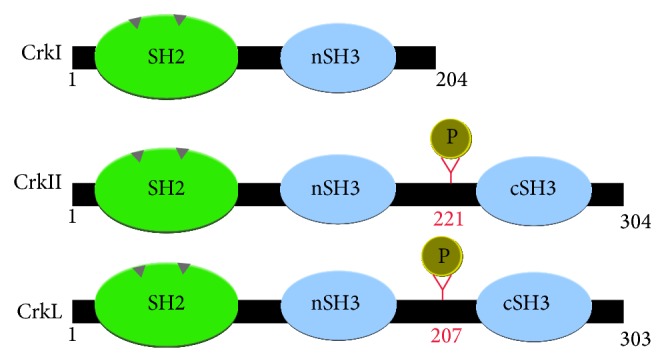
Schematic representation of Crk adaptor protein structure.

Crk proteins are prototypical adaptors (i.e., absence of enzymatic activity) consisting of Src homology 2 and Src homology 3 (SH2, SH3) domains. They possess one N-terminal SH2 domain, followed by one SH3 domain in the case of CrkI. However, CrkII and CrkL have two SH3 domains named according to whether they are more N- or C-terminally located, nSH3 and cSH3, respectively (Figure 1). The predicted molecular masses are 40 and 36 kDa for CrkII and CrkL, respectively, and 28 kDa for CrkI. They undergo posttranslational modifications as discussed below.

## 4. SH2 Domain

The SH2 domain is an approximately 100 amino acid module with intrinsic folding capacity. Structural studies performed with the Src kinase determined that SH2 domains have two binding surfaces and consequently bind simultaneously to a phosphorylated tyrosine (pTyr) and to motifs containing specific residues located C-terminal to the pTyr (residues +1 to +5). The first pocket contains highly conserved arginine and histidine residues whose mutations abrogate phosphate binding [25]. The second binding surface is more variable and is presumed to confer binding specificity (the specificity pocket; reviewed in [26, 27]). It is generally accepted that the consensus-binding motif for the SH2 domain of Crk proteins is pY-x-x-L/P.

**Figure 2:**
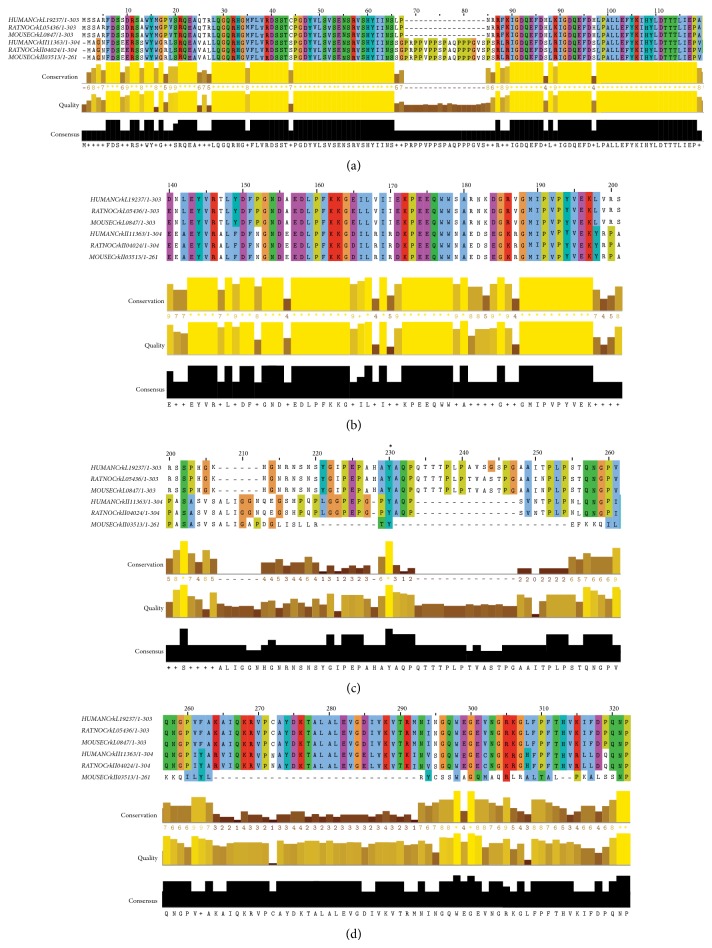
Crk alignments based on NCBI sequences by using the Clustal Omega program from the European bioinformatics institute (EMBL-EBI) (http://www.ebi.ac.uk/Tools/msa/clustalo/). (a) SH2 domains. Note the PRR in CrkII. (b) nSH3 domains. (c) Linker region containing the regulatory tyrosines (indicated by an asterisk). (d) cSH3 domains. Note the low homology of mouse CrkII.

A closer look into the structure of the SH2 domain of CrkI and CrkII shows that, compared with CrkL, they possess an extra stretch of 17 amino acids that contains a proline-rich region (PRR; PPVPPSPAQPPPGVSPS; Figure 2). This SH2-confined PRR has been shown to bind the SH3 domain of Abelson murine leukemia kinase (Abl) [28]. The SH2 domains of CrkII and CrkL share 82% homology. Remarkably, and unlike SH3 domains, SH2 domain containing proteins are apparently missing in prokaryotes [27].

## 5. SH3 Domain

SH3 domains are globular domains composed of approximately 60 amino acids that bind to PRRs in proteins with a generic PXXP core consensus and additional specificity-determining residues in the proximity, including a positively charged arginine or lysine residue. According to the orientation of the motif, SH3 domains have been classified into class I (+XXPXXP) and class II (PXXPX+) [29]. The nSH3 of CrkII and CrkL share 70% homology. In general, the nSH3 of Crk proteins binds polyproline class II motifs, although there are exceptions, such as the binding of CrkL nSH3 to the downstream of Crk 2 (DOCK2) protein that uses a bipartite motif [30].

CrkII and CrkL each contain a cSH3 defined by its inability to bind to classic polyproline type II (PPII) motifs (reviewed in [31]). Nuclear magnetic resonance (NMR) spectroscopy studies demonstrated that the cSH3 domain adopts the standard SH3 fold encompassing a five-stranded beta barrel; however, its binding surface contains several polar residues (such as glutamine and histidine) which suggests it may not bind typical PXXP ligands or that it may bind them with reduced affinity [32]. Thus, only one interaction has been described so far with the major nuclear export receptor named chromosome maintenance region-1 Crm1/exportin [33]. In fact, the binding site contains a nuclear localization signal (amino acids 256-266 in the human sequence). Therefore, the cSH3 domain allows for the nuclear export of CrkII and CrkL (see the nuclear import/export section).

## 6. Regulation

The nSH3 and cSH3 domains are bound by an approximately 50-residue-long linker (spacer) region that contains the so-termed regulatory tyrosines, Y221 in CrkII and Y207 in CrkL. It was proposed early on that the phosphorylated Y221 of CrkII could bind to its own SH2 domain [34]. Later, such a mechanism was also demonstrated for CrkL [35] and further confirmed using a fluorescence resonance energy transfer (FRET) biosensor ([36]; see below). CrkI regulation must be different because it lacks the regulatory tyrosine, a characteristic that was long ago presumed to be associated with its greater transforming activity [37].

The CrkI and CrkII structure were studied using small-angle X-ray scattering (SAXS) and nuclear magnetic resonance (NMR) spectroscopy ([38], commented on in [39]). The authors reported that residues 224–237, which are missing in CrkI, constitute an important regulatory element in CrkII called the inter-SH3 core (ISC). The ISC establishes contacts with the SH2 and SH3 domains to assemble CrkII into a compact structure in which the binding site of SH2 is exposed, but the SH2 domain masks the nSH3 domain. However, the cSH3 domain also contributes to the stability of the structure. It was also proposed that phosphorylation of the regulatory tyrosine not only blocks the SH2 domain, but also hides the binding surface of the nSH3 domain, generating a fully inhibited molecule. By contrast, CrkI has an extended structure in which both SH2 and nSH3 are freely accessible for interactions with target proteins.

*cis-trans* isomerization of P238 in chicken CrkII mediated by peptidyl-prolyl-*cis-trans* isomerases (PPIases) was proposed to be another regulatory element. In the* cis* conformation the nSH3 binding surface would be blocked by P238 and other residues from the cSH3 acting as a “reversible lid” ([40]; reviewed in [41]). However the sequence around the corresponding proline in human CrkII and CrkL (P237) is not well conserved, making the existence of such a regulatory mechanism less probable in human Crk [31]. A recent NMR spectroscopy study ([42]; discussed in [43]) pointed towards a distinct regulation for CrkL (see below).

## 7. Crk and Abl Kinases: Mutual Regulation

The Abl family of kinases, named after the Abelson murine leukemia virus (*v-Abl*) oncogene, comprises Abl and Arg (*Abl-related* gene; also known as Abl2). This family of nonreceptor tyrosine kinases has a modular structure, which includes one SH2 and one SH3 domain. They have a complex regulation and they play essential roles in regulating the actin-cytoskeleton, as indicated by the presence of an actin-binding domain in their C-terminus (reviewed in [44]).

The functional relationships between Crk and Abl are bidirectional (reviewed in [45]). The regulatory tyrosine of Crk adaptors is phosphorylated by the Abl family kinases [35]. The Crk nSH3 domain interacts with PRRs in Abl and induces its transactivation [46], which results in phosphorylation of Crk. In addition, phosphorylation of Y251 within the cSH3 of CrkII promotes a similar Abl transactivation [47].

CML and some adult acute lymphoblastic leukemia (ALL) patients frequently present the so called Bcr-Abl fusion protein, generated by a reciprocal chromosomal translocation (t(9;22)(q34;q11)) that originates from the “Philadelphia chromosome” [48]. The chimeric protein generated possesses a prominent disease-associated kinase activity that phosphorylates CrkL.

An elegant recent NMR spectroscopy study has uncovered a different global organization for CrkII and CrkL that would explain the predilection of the Bcr-Abl fusion protein for CrkL over CrkII [42, 43]. CrkL forms a constitutive complex with Bcr-Abl. Moreover, binding of phosphorylated Tyr207 (or any other phosphotyrosine) to the SH2 domain has no effect on the nSH3 that is still available to bind Bcr-Abl. In contrast, the association of CrkII with the kinase is repressed in at least two of the four proposed conformational states of the protein.

## 8. Crk Nuclear Import/Export

Although Crk adaptor proteins can enter the nucleus, the mechanisms governing their nuclear import/export are not well characterized. These proteins seem not to have a cognate nuclear localization signal (NLS); therefore, it is thought that they are imported through their interaction with other proteins that contain an NLS [49]. On the contrary, they have a typical nuclear export signal (NES) located in the cSH3, as previously mentioned. It has been reported that the translocation of CrkII to the nucleus is mediated by the interaction of the nSH3 domain with the cell cycle protein Wee1, DOCK180, or Abl. For example, in the complex formed by WeeI-CrkII, the NES of the latter is masked, keeping Crk in the nucleus, where the complex has pro-apoptotic functions.

CrkL nuclear export is mediated by the interaction with CrmI/exportin [33]. The cSH3 domain of CrkL can exist in monomeric or dimeric conformations in which partial unfolding of the domain exposes the NES [50]. A CrkL and CrmI complex is then formed that can be exported out of the nucleus. It is possible that CrkII shares this mechanism, taking into account the homology (93%) between their cSH3 domains. In addition, type I interferons signal for STAT5 phosphorylation, allowing the interaction with the CrkL SH2 domain [51, 52]. The CrkL-SH2 domain-phospho-STAT5 complex can translocate to the nucleus where it binds to the promoter region of c-Abl or Bcr-Abl in CML cells [52].

## 9. Crk Signaling Complexes

The current paradigm in the signaling of Crk adaptors is that interactions through the nSH3 are constitutive, while binding to the SH2 domain is primarily inducible. In other words, their SH2 domain senses pathway activation by upstream tyrosine kinases. However, phosphotyrosine signaling is now envisioned as more dynamic [53], with complex formation depending on protein availability, affinity of the implicated domains, post-translational modifications, and other factors.

CrkII itself and some of its key partners are focal adhesion (FA) proteins. FAs are multiprotein complexes containing plasma membrane-associated integrins that link the extracellular matrix and the actin cytoskeleton (reviewed in [54, 55]). Two of the first binding partners described for Crk were the p130 Crk-associated substrate (p130Cas; [56, 57]; reviewed in [58, 59]) and paxillin [60]. p130Cas is an adaptor protein whose tyrosine phosphorylation provides binding sites for the Crk SH2 domain and its association via the nSH3 domain with DOCK180, a guanine nucleotide exchange factor (GEF) that switches the small GTPase Rac1 to the GTP-bound active state [61].

FA formation and dynamics are challenging subjects to investigate because many different molecular assemblies of proteins are formed that are difficult to dissect. FAs are enriched in kinases, such as focal adhesion kinase (FAK) and Src family kinases (SFK). FAK is recruited to the cytoplasmic domain of integrin receptors. In one of the most studied cell adhesion pathways, a FAK-Src complex is formed that promotes paxillin and p130Cas phosphorylation at FAs. The adaptor protein p130Cas contains binding-sites not only for Crk, as mentioned above, but also for FAK, Nck, and Src.

Many proteins found at FAs participate in the formation of other “adhesive structures” such as the so-called “phagocytic cup.” Ig-opsonized pathogens are phagocytosed by professional phagocytes, mainly macrophages and neutrophils, as well as by M cells in the intestine, in a process that requires remodeling of the actin cytoskeleton through the action of the small GTPase Rac. CrkII and DOCK180 were found to accumulate at the phagocytic cup. Using Crk mutants and Crk siRNA, it was proposed that CrkII is required for DOCK180 recruitment, Rac activation, and pathogen engulfment [62]. The possible roles of Crk proteins in several human malignancies have remained elusive because studies have failed to correlate Crk expression levels with cancer progression [63–65]. There are several excellent reviews on the signaling of Crk proteins [9, 66].

## 10. Crk and Bacterial Invasion

Numerous reports indicate that Crk may contribute to bacterial pathogenesis in a variety of ways, including helping bacteria to enter host cells or serving as “targets of bacterial toxins that disrupt essential cellular functions” [9]. On one side, Crk adaptors have been implicated in lamellipodia and ruffle formation, in part because of their action over FAs [67] and, on the other side, in phagocytosis by immune cells [62]. It makes sense to think that intracellular pathogens would benefit by stimulating their invasion by promoting the first process, while they would benefit by inhibiting the second process to avoid their destruction by phagocytic immune cells. Thus, a common theme in bacterial invasion of nonphagocytic epithelial cells is the induction of ruffles at the site of bacterial attachment. The accumulation of bacteria along with the remodeled cytoskeleton at the site of entrance is very frequently referred as “foci.” This mechanism of entrance is called “the triggering mechanism” [68], in which we will focus with respect to Crk adaptors, as it is employed by various Enterobacteriacea members (e.g.,* Salmonella* and* Shigella*). On the contrary,* Yersinia* uses “the zipper mechanism.”

### 10.1. *Salmonella*

*Salmonella enterica* serovar Typhimurium targets intestinal epithelial cells that are normally nonphagocytic. Despite this,* Salmonella* is able to induce its phagocytic uptake after bacterial attachment to the intestinal epithelium [69]. Invasion is mediated by a type III secretion system (T3SS) that injects bacterial effector proteins directly into the host cytosol [70].* Salmonella *T3SS effectors directly target and manipulate host signaling pathways involved in actin filament assembly and cytoskeletal rearrangement leading to bacterial entry [71, 72].* Salmonella* invasion utilizes several pathways that converge on Crk: on one side the bacteria manipulate the Fak-p130Cas-Crk axis and on the other side the Abl/Arg-Crk signaling pathway.

To stimulate phagocytic uptake by host cells,* Salmonella* induces the assembly of FA-like complexes that lack integrins but require the recruitment of FAK and p130Cas [73]. FAK appears to act as a structural scaffold, as its kinase domain is not required. However, its C-terminal proline-rich motif, through which it interacts with p130Cas, is required. In addition, the p130Cas-CrkII interaction appears to be functionally important for* Salmonella*-induced cytoskeletal rearrangement. Thus, p130Cas^−/−^ cells complemented with p130Cas lacking the Crk-binding domain were impaired for invasion as compared with p130Cas^+/+^ cells. These data suggest that FAK, p130Cas, and Crk work in concert to regulate* Salmonella* invasion [73].

Another important strategy used by* Salmonella* is the manipulation of Abl kinases to promote lamellipodia formation through activation of the WASP family verprolin homologous protein (WAVE) complex [74], also called WAVE regulatory complex (WRC). WAVE proteins are actin-nucleation promoting factors (NPFs) that activate the Arp2/3 complex mainly at sites of lamellipodia formation (reviewed in [75]). Their regulatory mechanism is controversial, but it seems that activation of the WAVE2 complex requires simultaneous interactions with prenylated Rac-GTP and acidic phospholipids, as well as a specific phosphorylation [76, 77].

The role of Abl/Arg kinases and Crk phosphorylation has been studied by Casanova's group [19]. Abl is recruited to the site of bacterial invasion (reviewed in [72]). In MEFs either lacking Abl/Arg, or HeLa and MDCK cells treated with an Abl inhibitor (Imatinib), bacterial invasion efficiency was greatly reduced, as compared with untreated cells. It was proposed that the adaptor protein CrkII associates with Abl during infection, as evidenced by the presence of CrkII at the site of active* Salmonella* internalization where it colocalized with F-actin.* Salmonella* infection led to increased phosphorylation of CrkII, while CrkII phosphorylation deficient variants block* Salmonella* entry. Furthermore,* Salmonella* infection led to increased phosphorylation of the Abelson-interacting protein (Abi1) [19], a component of the WAVE2 complex. Interestingly, it has been proposed that Crk competes with Abi1 for binding to activated Abl, through interaction of their SH3 domain with the PRR in the kinase [45]. Thus, it is clear that both the Fak-p130Cas-Crk and Abl/Arg-Crk pathways make important contributions to* Salmonella* invasion. Because these pathways have largely been studied independently of each other, it will be interesting to determine if they are indeed interconnected.

### 10.2. *Shigella*

Like* Salmonella*,* Shigella flexneri* enters intestinal epithelial cells, which are not inherently phagocytic, and causes bacillary dysentery in humans.* Shigella* T3SS effectors interact with host proteins to induce dramatic rearrangements of the host actin cytoskeleton to form actin-rich extensions, similar to lamellipodia, which engulf the bacterium [78, 79]. However, in contrast to* Salmonella*, which replicates and survives inside a special compartment (the* Salmonella*-containing vacuole),* Shigella* remains in the cell cytoplasm [80]. Thus, although their invasion shares key components, including Crk, distinct host proteins seem to be used by these pathogens (e.g., cortactin).

*Shigella *stimulates the tyrosine phosphorylation of some host cell proteins, including Crk adaptors. Abl- and Arg-deficient MEFs have a dramatic decrease in intracellular bacteria after* Shigella* infection, as compared with WT cells. Similar to* Salmonella* infection, treating cells with Imatinib (which was developed to inhibit the kinase activity of the BCR-Abl fusion protein in Philadelphia-positive CMLs) reduced* Shigella* invasion compared with untreated cells, indicating that efficient* Shigella* infection requires Abl and Arg kinase activity [81]. It was reported that* Shigella* uptake promotes phosphorylation of Crk, indicating that the Abl-Crk module participates in* Shigella *invasion of host cells. In addition, a Crk-phosphorylation deficient mutant (CrkII-Y221F) showed a significant decrease in Rac and Cdc42 activation suggesting that the activation of these GTPases is at least in part mediated by Crk phosphorylation [81].

Cortactin, a type II NPF that activates the actin related protein (Arp) 2/3 complex and regulates the neural Wiskott-Aldrich syndrome protein (N-WASP) activity [82], is recruited to the site of* Shigella* entry and is tyrosine-phosphorylated in a Src kinase-dependent manner [83]. Bougnères et al. reported that, during* Shigella* invasion, Src phosphorylates cortactin and then phosphocortactin localizes to the site of* Shigella* invasion. Furthermore, it was proposed that phosphocortactin association with Crk promotes* Shigella* entry [84]. Contrary to* Shigella*, cortactin expression downregulation by RNA interference does not inhibit* Salmonella* invasion, although the protein is recruited to the sites where the bacteria invade [85].

Unc119 was initially identified as an adaptor protein that activates specific SFK members, such as Lyn [86]. Interestingly, Unc119 blocks* Shigella* invasion by inhibiting Abl/Arg tyrosine kinases, which results in Crk phosphorylation downregulation [87]. While for* Salmonella*, the WAVE complex has been well studied, relatively little data are available for* Shigella*, while the opposite is true for cortactin. Therefore, potential future studies could examine the roles of these respective proteins to determine if they are indeed important to* Shigella *and* Salmonella* invasion, respectively.

### 10.3. *Yersinia*

*Yersinia pseudotuberculosis* and* Y. enterocolitica* are enteric pathogens that cause infections which are usually self-limiting, in contrast to* Y. pestis*, the causative agent of bubonic plague (reviewed in [88]).* Yersinia* species deliver virulence proteins (Yop effectors) directly into the host cell via the T3SS [89].* Yersinia* spp. utilize a *β*1-integrin-mediated zippering mechanism for bacterial uptake in an actin-dependent process. Uptake is mediated by the interaction between invasin, a bacterial transmembrane protein, and *β*1-integrins on the host cell surface. The invasin-integrin association initiates a signaling cascade, characterized by tyrosine phosphorylation, leading to activation of the Rho GTPases Rac1 and Cdc42, possibly to promote the activation of N-WASP and the Arp2/3 complex [90] for the formation of actin projections enabling bacterial invasion.

Crk-p130Cas signaling was implicated in* Y. pseudotuberculosis* uptake [91]. Overexpression of Crk containing mutations (R38V, W169L) to block Crk-SH2 or Crk-SH3 domain binding to target proteins decreased bacterial uptake, indicating that Crk is important for* Yersinia* invasion of epithelial cells. Furthermore, p130Cas-Crk complex formation was induced in response to infection, which was coupled to Rac1 activation. However, the existence of a Fak-dependent and p130Cas-Crk independent pathway for* Yersinia* uptake has also been proposed [92].

In contrast to invasin, which binds directly to *β*1-integrin receptors with high affinity, YadA is a* Y. pseudotuberculosis* outer membrane adhesin that binds integrins indirectly through the extracellular matrix (ECM). Hudson et al. characterized the mechanisms by which invasin and YadA promote adherence and phagocytic signaling events in macrophages and studied how ECM proteins differentially influence their ability to bind integrins. At low ECM concentrations, invasin binds directly and with high-affinity to *β*1-integrin to induce integrin clustering and to stimulate integrin-dependent phagocytosis, through FAK-p130Cas-Rac1 signaling. However, at high ECM concentrations, indirect YadA binding to *β*1-integrin predominates, which also promotes adherence to and entry into host cells [93]. Together, these data suggest that the host cell response to* Yersinia* infection is likely influenced not only by the expression levels of invasin and YadA during infection, but also by the extracellular environment at the infection site.

It is noteworthy that Crk is implicated in the triggering mechanism for* Salmonella* and* Shigella*, and in the zipper mechanism of invasion for* Yersinia*. This may not be surprising because Crk is implicated in cytoskeletal remodeling via Rac GTPase and possibly WAVE regulation, whereas it is also involved in FA remodeling through integrin signaling.

## 11. EPEC

Enteropathogenic and enterohemorrhagic* Escherichia coli* (EPEC and EHEC, resp.) adhere to intestinal epithelial cells and deliver proteins into the host via the T3SS, resulting in microvilli effacement and intimate attachment to cells, forming the so-called attaching and effacing (A/E) lesions. Because most of the data obtained to date regarding Crk signaling has been from studies of EPEC, rather than EHEC, we will primarily focus on EPEC in this review. It will be interesting in the future to determine to what extent these data are translatable to EHEC.

Among the effector proteins delivered is the translocated intimin receptor (Tir), which is inserted into the host cell plasma membrane where it binds intimin, located on the outer membrane of EPEC [94]. The Tir-intimin interaction activates signaling events that are required for A/E lesion formation. The accumulation of a dense material identified as actin was noticed in early studies. The resulting actin-rich structure is now referred to as a pedestal (reviewed in [95]).

EPEC Tir is phosphorylated on residue Y474 [96] redundantly by Abl/Arg [97] and SFK tyrosine kinases [98], which is essential for actin polymerization and pedestal formation [96]. Besides tyrosine phosphorylation, Tir is phosphorylated on serine residues (S434 and S463). This phosphorylation has been correlated to increases in apparent molecular mass and efficient pedestal formation [99]. It is thought that these shifts in apparent molecular mass indicate changes in Tir structure that enable tyrosine phosphorylation and/or promote Tir insertion into the plasma membrane [99, 100]. Phosphorylated Y474, within the C-terminal cytoplasmic domain of Tir, recruits the host cell adaptor proteins non-catalytic tyrosine kinase (Nck) 1 and 2 (collectively referred to as Nck). Nck in turns recruits N-WASP [101], an NPF member of the WAS family of proteins that promote actin polymerization by activating the Arp2/3 complex [102, 103]. N-WASP presents a closed inactive conformation mainly due to intramolecular autoinhibitory interactions that involve the C-terminal acidic domain and the GTPase-binding domain (GBD). N-WASP requires the interaction with other proteins through its GBD or proline-rich domain (PRD) and possibly posttranslational modifications to be fully active [75].

The current paradigm establishes that the major pathway to actin polymerization in typical EPEC infections takes place through phosphorylation of Tir Y474, and subsequent formation of a Tir-Nck-N-WASP complex to promote actin polymerization by the Arp2/3 complex [104]. This proposed signaling pathway has been based mainly on studies using Nck-deficient MEFs, thus their low efficiency in pedestal formation was presumed to reflect a lack of N-WASP activation [101, 105–107]. It is also generally accepted that phosphorylated Tir Y454 promotes a secondary Nck-independent pathway for actin nucleation [107].

However, infection studies with human intestinal tissue using an EPEC Tir Y454F,Y474F variant also lead to actin nucleation and pedestal formation [108]. Likewise, A/E lesion formation and N-WASP recruitment was unaltered when the equivalent Tir variant from* Citrobacter rodentium* (Tir_CR_ Y471 and Y451) was used [109]. These* in vitro* versus* in vivo* contradictory results might be explained at least in part by our recent unexpected findings indicating that Nck adaptors possibly have a subsidiary function in activating N-WASP during pedestal formation by EPEC (Nieto-Pelegrin et al,* Cell Adhesion and Migration, In press*). We found decreased levels of translocated Tir within Nck1/2-deficient MEFs that were corroborated in HeLa cells with down-regulated expression of Nck by siRNA.

## 12. EPEC Manipulation of Focal Adhesion Proteins

In the early 1960s it was reported that EPEC induces epithelial cell shedding in the intestine [110] which may contribute to diarrhea. This effect was later reproduced in studies with cell lines, including epithelial cells (HeLa, Caco-2) and fibroblasts (DU17; [111]). It was observed that EPEC induces cell detachment from the substratum of the infected host cells mainly by modifying FAs, which were reduced in number and redistributed to the cell periphery. Furthermore, this T3SS dependent-detachment was correlated with FAK dephosphorylation and thus, in FAK^−/−^ fibroblasts, detachment could not be detected [111]. However, the molecular mechanism underlying cell detachment induced by EPEC remained elusive, until it was recently found that the non-LEE-encoded EspC, a serine protease injected by EPEC, is responsible for FAK dephosphorylation and its subsequent degradation, as well as for the degradation of other FA proteins such as paxillin [21].

Related to that, an increase in FAK dephosphorylation by the* Shigella* late T3SS effector OspE has been reported [112]. OspE interacts with integrin-linked kinase (ILK) and blocks focal adhesion disassembly. ILK is a central adaptor (a pseudokinase) recruited to *β*1-integrin tails in FAs that mediates the communication between cells and the ECM. Future work will undoubtedly address whether the anticell lifting effect mediated by OspE binding to ILK, demonstrated for* Shigella*, applies to the OspE orthologs found in EPEC, EHEC, and* Salmonella* [113, 114]. As for EHEC, the EspO1-2 effectors are homologous to* Shigella* OspE, and through EspM, seem to regulate RhoA GTPase activity to stabilize FAs [115]. Since Crk adaptors participate in FAs signaling, future studies should aim to address their role in FAs manipulation by these pathogens.

Using immunofluorescence staining, Goosney et al. localized other FA components besides FAK, including p130Cas, vinculin, and CrkII [116]. CrkII localization within pedestals was shown to be dependent on the phosphorylation of Y474 of Tir, although it should be noted that the Y474F variant induces a very limited number of pedestals. In addition, Crk proteins interact with N-WASP via SH3-PRR interactions in smooth muscle cells [117]. However, it still remains unknown whether Crk-N-WASP interaction would activate the latter to promote Arp2/3 complex dependent actin polymerization.

These and other unanswered questions prompted us to investigate the role of Crk adaptors in pedestal formation by EPEC [20]. Unexpectedly, we found that Crk isoforms act as redundant inhibitors of pedestal formation. Thus, Crk expression downregulation by siRNA in HeLa cells or the absence of individual Crk isoforms within CrkI/II or CrkL K.O. MEFs did not alter the number of pedestals formed in infected cells. On the contrary, inhibition of the three Crk isoforms in HeLa cells resulted in a significant increase in pedestal number. Similar results were found in CrkI/II or CrkL K.O. MEFs with knock-down expression by siRNA of the remaining isoform. Moreover, we found that Crk SH2 domain binds Tir through Y474 and competes with the binding of the Nck SH2 domain to Tir, thus inhibiting its recruitment and subsequently, N-WASP activation. In view of our findings, we proposed that Crk adaptors might inhibit actin polymerization at pedestals by competing with Nck activation of N-WASP [20].

## 13. Do Crk Adaptors Have a Role in Innate Immunity Signaling Manipulation by EPEC?

Several EPEC and EHEC T3SS effectors have been found to inhibit the innate immune response of intestinal epithelial cells by altering the activity of various components of the proinflammatory NF-*κ*B signaling pathway. This is the case for Tir, whose interaction in the cytoplasm with the TNF-alpha receptor-associated factor (TRAF) adaptor proteins induces their degradation by, at the moment, an undefined mechanism [118]. EPEC Tir contains ITIM motifs that interact with SHP-1, a host tyrosine phosphatase, implicated in immune downregulation. Tir binding to SHP-1 was found to promote the association of SHP-1 with TRAF6 and inhibit TRAF6 ubiquitination, thus altering immune signaling [119]. While Tir is known to bind Crk [20], this interaction is not necessarily implicated in the phenotypes described above, and awaits further experimentation.

Several years ago it was discovered that the ribosomal protein S3 (RPS3) interacts with the p65 subunit of NF-*κ*B in the nucleus to increase the affinity of the NF-*κ*B complex for a subset of gene promoters [120]. The non-LEE-encoded effector NleH1 inhibits I*κ*B-kinase-*β* (IKK*β*) phosphorylation of RPS3 S209, preventing RPS3 nuclear translocation [121] and thus reducing NF-*κ*B activity in infected cells [122]. Wan et al. reported that the Ser/Thr kinase activity of NleH1 is essential to inhibit NF-*κ*B activation, yet neither RPS3 nor IKK*β* appeared to be the target for NleH1 kinase activity [121]. Using an* in vitro* kinase array, CrkL was identified as an NleH1 kinase substrate and was also found to interact with IKK*β* [22]. Downregulation of CrkL expression using siRNA prevented NleH1 inhibition of NF-*κ*B activity, suggesting that CrkL may act as an adaptor protein, possibly by recruiting NleH1 to the IKK*β*-RPS3 complex to prevent RPS3 phosphorylation and subsequently inhibit NF-*κ*B activation [22]. It is currently unknown if this adaptor function in innate immunity applies only to CrkL or might also involve CrkII, as these proteins often have redundant functions. It is also unknown if CrkII/CrkL activities might influence the action of other EPEC/EHEC effectors with anti-inflammatory functions.

## 14. Crk Adaptors as Possible Targets in the Treatment of Enterobacteriaceae Infections

Fortunately, there is a lot of active research in the area of pharmacological inhibition of Crk, due to the oncogenic role of Bcr-Abl fusion-protein in CML. Microbiologists could exploit much of this work for future research. As mentioned above, the Abl inhibitor imatinib significantly reduced* Salmonella* and* Shigella* infection of cells, an observation that appears promising regarding its potential use in animal models. Evolving knowledge on microRNAs (such as miR-126, [123]) could be taken into consideration as a possible way to prevent infection of* Salmonella*,* Shigella*, and* Yersinia*.

On the contrary, according to* in vitro* studies [97], chemical inhibition of Abl/Arg might be expected to fail in the case of preventing EPEC infection. However, the discrepancies found between the different* in vitro* and* in vivo* models, as previously mentioned, will dictate careful reassessment. The subject gets even more complicated because, as discussed above, on one side, CrkII and CrkL block Tir signaling to achieve pedestal formation, which could be explained by the recent finding that Crk adaptors function in a heterocomplex, as reported to occur during podocyte morphogenesis [124]. Therefore, concomitant inhibition of CrkII and CrkL might result in an increase in pedestal formation, which would seem to favor bacterial adhesion. However, individual CrkL expression downregulation was sufficient to block NleH1-mediated inhibition of NF-*κ*B, which could imply a different mechanism of action and a direct role of Crk adaptors in promoting innate immune responses. Clearly, more research is needed to gain further insight into how Crk adaptors participate in the host response to Enterobacteriacea infections.

## 15. Concluding Remarks

**Figure 3:**
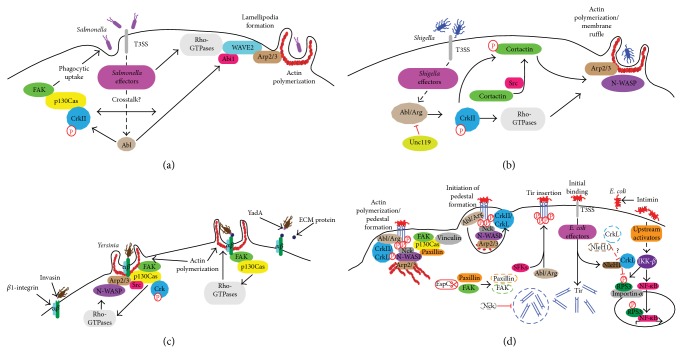
Roles of Crk proteins in bacterial pathogenesis. (a)* Salmonella enterica* serovar Typhimurium manipulates Abl kinase phosphorylation of Crk adaptors to gain entry into the host cell.* S. Typhimurium* activates Abl kinase via T3SS effectors. Abl phosphorylates CrkII regulatory tyrosines. FAK and p130Cas are recruited to the FA-like complex where p130Cas interacts with CrkII, leading to cytoskeletal rearrangement and uptake of the bacterium. Abl activation by* Salmonella* effectors may also result in WAVE2 complex activation, in part through phosphorylation of Abi1. The WAVE2 complex is necessary for activation of Arp2/3 to induce actin polymerization at the site of lamellipodia formation. WAVE2 activation also requires interaction with Rho-GTPases (e.g., Rac).* Salmonella* effectors may also directly activate Rho-GTPases. It is unclear whether crosstalk between the two pathways exists (adapted from [19]). (b)* Shigella* T3SS effectors activate Abl/Arg to phosphorylate Crk adaptors. Abl/Arg phosphorylates and activates CrkII, which regulates the activity of Rho-GTPases (e.g., Rac and Cdc42). Cortactin is phosphorylated by Src and phosphocortactin interacts with CrkII. Cortactin can contribute to activation of N-WASP and the Arp2/3 complex, resulting in actin polymerization and membrane ruffling. Rho-GTPases can also activate Arp2/3 and N-WASP. On the contrary, Unc119 blocks Abl/Arg phosphorylation of Crk. (c)* Yersinia* uptake is mediated by *β*1-integrin signaling to Crk adaptors. The p130Cas-Crk interaction is promoted during* Yersinia* invasion. FAK and Src are also recruited to this complex. The p130Cas-Crk complex formation is coupled to Rho-GTPase (e.g., Rac) activation, leading to activation of N-WASP, actin polymerization, and bacterial entrance. Alternatively,* Yersinia* YadA can bind to host cell integrin indirectly through ECM proteins (e.g., fibronectin). This activates a signaling cascade involving FAK, p130Cas and Rho-GTPases, which ultimately lead to actin remodeling and* Yersinia* uptake. At low ECM concentrations, the invasin-integrin model predominates. At high ECM concentrations, the YadA-ECM-integrin model predominates.* Yersinia* likely uses a combination of invasin and YadA binding to gain entry into the cell. (d) Crk adaptor proteins are targeted by several EPEC effectors during infection. Tir is inserted into the host cell membrane where binds intimin on the bacterial surface, resulting in intimate attachment of* E. coli* to the host cell. Abl/Arg and Src family kinases (SFKs) phosphorylate Tir (“Tir insertion”), resulting in the recruitment of Nck. Nck recruits and activates N-WASP leading to Arp2/3 complex-mediated actin polymerization and pedestal formation. Abl/Arg phosphorylates CrII/CrkL (“initiation of pedestal formation”; see [20] for details). Tir levels are reduced in the absence of Nck (dashed circle; Nieto-Pelegrin et al, in press). Other FA components localize to pedestals (e.g., FAK, p130Cas, vinculin, and paxillin; “Actin polymerization/Pedestal formation”), though some are degraded by the EspC effector (dashed lines; [21]). During infection, the transcriptional regulator, NF-*κ*B, becomes activated and translocates to the nucleus. In addition, RPS3 is phosphorylated by IKK*β* and translocates to the nucleus via importin-*α*, where RPS3 acts as a “specifier” for NF-*κ*B to select for and regulate a specific subset of innate immune response genes. The effector NleH1 interacts with CrkL and subsequently prevents the phosphorylation of RPS3, thus blocking RPS3 nuclear translocation and inhibiting NF-*κ*B activation and the innate immune response. In the absence of CrkL (dashed lines), NleH1 cannot block RPS3 translocation to the nucleus. Model partially adapted from [22]. Other translocated effectors include mitochondrial associated protein (Map) and EPEC-secreted proteins (Esp) H, F, G, and Z [23] that are encoded within a pathogenicity island termed the locus of enterocyte effacement (LEE) [24].

Much has been learned about the role of Crk adaptors in host-pathogen interactions (Figure 3). However many unanswered questions regarding their signaling pathways remain open to investigation, especially with regard to a detailed dissection of the molecular complexes that are formed. In addition, some of the pathways in which Crk adaptors have been implicated are still incomplete and disconnected. There is little doubt that future work should address some challenging matters of pathogen manipulation of cellular signaling. One of those key aspects is the temporal regulation of signaling. The picture gets complicated by the apparent redundancy in effector targets (i.e., the same cellular proteins can be targeted by different bacterial effectors), even more so when these targets are functionally pleiotropic proteins such as Crk adaptors. In our opinion, although Crk adaptors have been traditionally involved in the entrance of pathogens, thanks to recent studies, it seems that they will also be further implicated in innate signaling.

## Abbreviations

Abl: Abelson murine leukemia kinase
Arp: Actin-related protein
Crk: CT10 (chicken tumor virus number 10) regulator of kinase
CrkI/II^−/−^: CrkI/II-deficient MEFs
CrkL^−/−^: CrkL-deficient MEFs
CrkL: Crk-like
Dock: Dedicator of cytokinesis
EPEC: Enteropathogenic* E. coli*
FA: Focal adhesion
F-actin: Filamentous actin
FAK: Focal adhesion kinase
LEE: Locus of enterocyte effacement
MEFs: Mouse embryonic fibroblasts
NCK: Noncatalytic tyrosine kinases (Nck) 1 and 2
NF-*κ*B: Nuclear factor kappaB
p130Cas: p130 Crk-associated substrate
PPIase: Peptidyl-prolyl cis-trans isomerase
PRR: Proline-rich region
SH2 and SH3: Src homology 2 and 3 domain
Src tyrosine kinase: Sarcoma tyrosine kinase.
